# Olfactory Function in Patients with Inflammatory Bowel Disease (IBD) Is Associated with Their Body Mass Index and Polymorphism in the Odor Binding-Protein (OBPIIa) Gene

**DOI:** 10.3390/nu13020703

**Published:** 2021-02-22

**Authors:** Giorgia Sollai, Melania Melis, Mariano Mastinu, Danilo Paduano, Fabio Chicco, Salvatore Magri, Paolo Usai, Thomas Hummel, Iole Tomassini Barbarossa, Roberto Crnjar

**Affiliations:** 1 Department of Biomedical Sciences, University of Cagliari, Monserrato, 09042 Cagliari, Italy; melaniamelis@unica.it (M.M.); mariano.mastinu@unica.it (M.M.); tomassin@unica.it (I.T.B.); crnjar@unica.it (R.C.); 2 Department of Medical Sciences and Public Health, University of Cagliari, Presidio Policlinico of Monserrato, 09042 Cagliari, Italy; danilo.paduano@libero.it (D.P.); Chicco.fabio90@gmail.com (F.C.); salvo10ms@libero.it (S.M.); paolo.usai@unica.it (P.U.); 3 Smell and Taste Clinic, Department of Otorhinolaryngology, TU Dresden, 01067 Dresden, Germany; thomas.hummel@tu-dresden.de

**Keywords:** smell, olfactory dysfunction, Crohn’s disease (CD) and ulcerative colitis (UC), odorant binding proteins (OBPs) gene, BMI, nutrition

## Abstract

Smell strongly contributes to food choice and intake, influencing energy balance and body weight; its reduction or loss has been related to malnutrition problems. Some patients with inflammatory bowel disease (IBD), mainly Crohn’s disease (CD) and ulcerative colitis (UC), are underweight, while others are overweight. Some studies suggest that changes in eating habits could be linked to specific disorders of the olfactory functions. We assessed the olfactory performance in 199 subjects (healthy control (HC) *n* = 99, IBD *n* = 100), based on the olfactory Threshold, Discrimination and Identification score (TDI score), measured with the “Sniffin’ Sticks” test. Subjects were genotyped for the *rs2590498* polymorphism of the *OBPIIa* gene. IBD patients showed both a slightly, but significantly, lower olfactory function and a higher BMI compared to HC subjects. Threshold (in both population) and Discrimination (in IBD patients) olfactory score were affected by the *OBPIIa* genotype. BMI was influenced by both health status and *OBPIIa* genotype. A lower olfactory function may delay the satiety sensation and thus increase meal duration and body weight in IBD patients. However, the AA genotype of the *OBPIIa* seems to “protect” IBD patients from more severe olfactory dysfunction.

## 1. Introduction

The olfactory system is traditionally responsible for the perception of volatile chemicals, odorous compounds or odorants, providing information from the external environment, and recently, it is becoming widely considered a sensor of the internal environment to the individual [1]. The sense of smell plays a critical role in various aspects of human life, and its important functions can be grouped into three broad categories: avoidance of environmental hazards, social communication and ingestive behavior [2]. Olfaction draws attention to warning signs, such as predators, fire, smoke, toxic substances or poisons and spoiled food [3,4]; it impacts reproductive behavior [5,6,7,8,9]; it affects interpersonal communication, such as between a mother and her baby [10]; it contributes to the localization, choice and intake of food, and it modulates appetite [2,11,12,13,14,15,16,17,18,19].

Human perception of odors differs among individuals, and this diversity is due to both environmental and genetic factors [20,21,22,23,24]. The variations in chemosensory sensitivity can be quite large, and individuals can have high sensitivity (hyperosmia), reduced sensitivity (hyposmia), general and/or partial inability to perceive odorants or a specific odor (general or specific anosmia) [22,23,25,26,27,28]. Olfactory deficits, present in about 12%–20% of the population, have a strong effect on the quality of life; people with olfactory dysfunctions report an increased number of domestic accidents, severe social insecurity, a tendency for depression and eating disorders [2,13]. In particular, people with olfactory impairment report having changed their eating behavior as they find food less flavorful and less enjoyable, have problems with cooking and often eat spoiled or burnt foods [12,29,30].

Progressive loss of smell is associated with the presence of chronic diseases, such as hypertension, neurodegenerative diseases (Parkinson’s, Alzheimer’s), depression and obesity [31,32,33,34]. Furthermore, recent studies have highlighted a relationship between olfactory function and autoimmune/inflammatory diseases, such as rheumatoid arthritis, psoriasis, myastenia gravis, Sjogren’s syndrome and inflammatory bowel diseases [35,36,37,38,39,40,41]. 

Crohn’s disease (CD) and ulcerative colitis (UC) are the major clinical manifestations of gastrointestinal diseases known as inflammatory bowel diseases (IBD) [36,42,43,44,45]. The precise etiology of IBD is not yet fully understood, but several studies have shown that interactions between genetic and environmental factors are associated with this pathogenesis [46,47,48,49,50,51]. Since the incidence of IBD is higher in Western countries and, in recent decades, it has also been increasing in countries, such as China and India, as a consequence of the industrialization and westernization of lifestyle, it is believed that environmental exposure is the factor of more significant risk for developing IBD [43,44,52,53]. Environmental risk factors in the pathogenesis of IBD patients include smoking, oral contraceptives, stress, appendectomy and dietary changes [54,55]. Among these factors, it seems that the diet has a prominent role, by way of a mechanism not well defined [56]. Sugihara and collaborators [43] report that the nutrients in the diet are known to have an impact on the health of individuals, through complex interactions with the intestinal immune system; therefore, a well-balanced diet is crucial for good health. In agreement, the consumption of foods low in fiber, high in refined sugars and animal fats has been observed to increase the risk of IBD [56,57,58]. Similar food preferences have been found in individuals with hyposmia (i.e., reduced olfactory function), who show a higher consumption of sugars, fats and salt to make meals more palatable and thus compensate for the less flavorful and enjoyable sensation resulting from the reduced olfactory sensitivity [12,30,36]. 

Given the importance that olfactory performance plays in the dietary behavior and food choices of individuals, the main objective of this study was to assess the olfactory function in IBD patients and in a control group of healthy subjects (HC), who were age- and gender-matched. Since both weight loss and weight gain have been observed in IBD patients [36,59] and reported as a consequence of a loss of olfactory function [30], we evaluated differences in body mass index (BMI) between IBD patients and HC subjects, also according to their olfactory function. Finally, as the physiological variations of olfactory performance can be attributed, at least partially, to the *rs2590498* (A/G) polymorphism of the gene encoding for human odorant binding protein (hOBPIIa) [24,60,61], we investigated the role of this polymorphism in the olfactory function of IBD patients and HC subjects, also according to their BMI.

## 2. Materials and Methods

### 2.1. Subjects

One hundred and ninety-nine Caucasian volunteers were recruited in the metropolitan area of Cagliari (Sardinia, Italy) and were divided into two groups. The first group was represented by the Inflammatory Bowel disease (IBD) patients (*n* = 100; 54 men, 46 women; age 51.2 ± 1.41 year), recruited for the study at the clinic of the Gastroenterology Unit of the University Hospital Company (AOU) Monserrato (CA), Italy and included Crohn’s disease (CD) (*n* = 44) and ulcerative colitis (UC) patients (*n* = 56); the second group included healthy control (HC) subjects (*n* = 99; 59 men, 40 women; age 47.3 ± 1.42 year) recruited by means of a public announcement at the local University, so as to have two populations matched for age (*χ2* = 4.33, *p* = 0.12) and gender (*χ2* = 0.64, *p* = 0.43). IBD diagnosis was obtained by a combination of clinical examination, endoscopy and radiology, and by means of the Crohn’s Disease Activity Index (CDAI) and Partial Mayo Score (PMS), the disease activity was evaluated for CD and UC patients, respectively [62]. All tested patients with CD and UC were recovering from the disease and were treated with cortisone or mesalamine or 5-ASA agents or monoclonal antibodies against TNF-α. BMI of IBD patients was stable and did not change over time due to disease remission conditions. For all HC subjects and IBD patients, exclusion criteria were diabetes, major systemic diseases, pregnancy or lactation, use of medicines interfering with smell perception, head trauma, sinusitis or nasal septum disorders. Prior to testing, controls and patients were asked if they had a cold or had any allergic reactions, in which case they were discarded. All subjects were free of perfumes and fasted for at least 2 hours prior to testing. 

The study was conducted in accordance with the Declaration of Helsinki and was approved by the Ethical Committee of the University Hospital of Cagliari. Before being tested, each subject was informed on the purpose of the study and the experimental procedures and was asked to sign an informed consent.

### 2.2. Olfactory Sensitivity Screening

The standardized “Sniffin’ Sticks” test battery (Burghart Instruments, Wedel, Germany) comprising three subtests for olfactory threshold (T-test), olfactory discrimination (D-test) and olfactory identification (I-test) was used to evaluate the orthonasal olfactory function of individuals [63]. This test is widely used for olfactory screening, and its validity, also in the health field, is internationally recognized. Odors were presented by means of felt-tip pens: the pen cap was removed, and the pen tip was then positioned under the nose, approximately 2 cm away from the nostrils for 3 sec. During the T-test and D-test, the subject was also blindfolded or otherwise asked to keep their eyes closed in order to prevent them from learning to identify the pen containing the odor by sight [63].

For the determination of the olfactory threshold, the experimenter had 48 pens available, divided into 16 triplets, each of them consisting of two pens containing a solvent and the third soaked in a growing concentration of n-butanol. Triplets were presented in ascending order until the subject identified, for two consecutive times in the same triplet, the pen containing n-butanol. This was the starting point and represented the first reversal, that is, the point where the triplets were presented in descending dilution order of n-butanol. Each time the subject failed to recognize the target pen, the dilution order with which the triplets were presented was reversed. The experiment ended when the seventh reversal was reached, and the threshold score was given by the average of the last four reversals. Additionally, for the determination of odor discrimination, the experimenter had 16 triplets available, each formed by two pens containing the same odor and one filled with a different one (target pen). The participant’s goal was to identify the target pen. The score obtained corresponds to the number of correct answers from 0 to 16. To determine the identification of odors, 16 pens were used containing as many odors familiar to the subjects. Each pen was associated with 4 possibilities from which the subject was required to choose. The score corresponds to the number of correct identifications from 0 to 16.

During the test, the experimenter compiled a protocol in which the scores obtained for each sub-test, age, gender, height, weight and BMI were reported. The sum of the scores obtained with the T-test, D-test and I-test gave the total Threshold, Discrimination and Identification (TDI) score, by which the subjects were classified for their general olfactory performance as normosmic or hyposmic. On the basis of the score obtained with the T-test, D-test and I-test, subjects could also be classified by olfactory threshold, olfactory discrimination and olfactory identification [64].

### 2.3. Genetic Analysis

The “QIAamp® DNA” Mini Kit (Qiagen srl, Milan, Italy) was used to extract the DNA from the saliva samples, in accordance with the instructions. Saliva samples (approx. 2 mL) were collected from each subject in DNA- and RNA-free Eppendorf Safe-Lock tubes the day they came to the laboratories to be subjected to the olfactory tests. The purity and concentration of the DNA were estimated by measuring the optical density at the wavelength of 260 and 280 nm, respectively. Subjects were genotyped for the rs2590498 (A/G) polymorphism of the OBPIIa gene, and DNA was amplified using custom TaqMan® SNP Genotyping Assay (Applied Biosystems by Life-Technologies Italia, Europe BV). The PCR reactions were run in duplicate in a StepOnePlus™ instrument (Applied Biosystems) using the following two primers: sense primer 5′-GCCAGGCAGGGACAGA-3′ and the antisense primer 5′-CTACACCTGAGACCCCACAAG-3′. Two TaqMan probes were drawn according to the OBPIIa gene (bold and underlined), probe/reporter 1: VIC- TCGGTGAC**A**TGAACC and probe/reporter 2: FAM-TCGGTGAC**G**TGAACC. After the PCR runs, the samples were read by a gene sequence reading system at 60 °C for 1 min, and the allelic discrimination was determined with sequence detector software (Applied Biosystems). Positive controls (whose polymorphism was already known) and no-template controls (NTC) were included in all reactions.

### 2.4. Data Analyses

IBD patients are represented as a single panel, because no differences were found between CD and UC patients for all statistical analyses.

Stepwise, multiple linear regression was used to determine the relative contribution of T, D and I scores as predictor variables of TDI score, in both HC subjects and IBD patients. The relative contribution of each significant variable and the semipartial correlations (sr) for each of them are reported in Table 2. 

One-way ANOVA was used to analyze the effect of the health status (HC or IBD) of the subjects on the score obtained with the T-test, D-test, I-test and their sum TDI.

Two-way ANOVA was used to test for a significant interaction between health status (HC or IBD) × OBPIIa genotype on the T, D and I scores. 

Two-way multivariate analysis of variance (MANOVA) was used to analyze differences of the T, D and I scores (within factors) according to the OBPIIa genotype and health status of the subjects (HC or IBD) (between factors).

Data were checked for the assumptions of homogeneity of variance and normality. Post-hoc comparisons were conducted with the Fisher’s least significant difference (LSD) test or Duncan’s test when the assumption of homogeneity of variance was violated [65,66]. Statistical analyses were performed using STATISTICA for WINDOWS (version 7.0; StatSoft Inc., Tulsa, OK, USA). P values < 0.05 were considered to be significant. 

Fisher’s Exact Test was used to analyze differences in the TDI, T, D and I olfactory statuses between HC subjects and IBD patients.

Differences in genotype distribution and allele frequencies at the *OBPIIa* locus between HC subjects and IBD patients were analyzed by using Fisher’s method (Genepop software version 4.2; http://genepop.curtin.edu.au/genepop_op3.html) [67].

## 3. Results

### 3.1. Olfactory Scores and Subjects Classification

**Figure 1:**
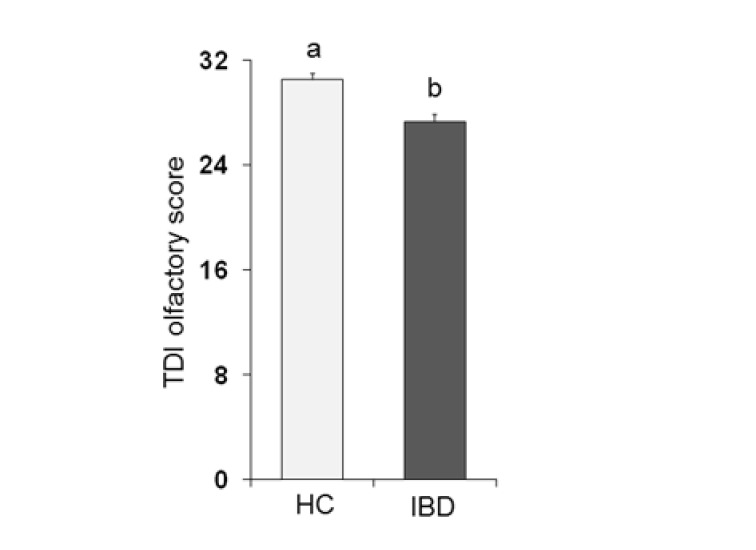
Mean (± SE) values of the Threshold, Discrimination and Identification (TDI) olfactory score determined in healthy control (HC) subjects (*n* = 99) and inflammatory bowel disease (IBD) patients (*n* = 100). Different letters (a and b) indicate a significant difference (*p* < 0.001, Duncan’s test).

Figure 1 shows the mean values ± SE of the total TDI olfactory score obtained from each population considered: HC subjects and IBD patients. One-way ANOVA showed that the TDI score obtained by HC subjects was significantly higher than that obtained by IBD patients (*F*_1,197_ = 22.75; *p* < 0.001). 

The percentage of subjects who were classified as normosmic or hyposmic for their TDI olfactory status in HC subjects differed from that determined for IBD patients (*χ2* = 5.499, *p* = 0.019) (Table 1). In detail, 60.61% (*n* = 60) and 39.39% (*n* = 39) of HC subjects were, respectively, normosmic or hyposmic, while 44% and 56% of IBD patients were normosmic or hyposmic, respectively.

Table 2 shows that the relative contribution of each subtest to the TDI score was significant for both HC subjects and IBD patients, albeit in a different way. Indeed, in HC subjects, the major contributor to the model was the T score (52.05%), secondly the D score (20.90%) and finally the I score (24.20%). Instead, in IBD patients, the major contributor to the model was the score obtained with the D-test (70.33%) and, to follow, the T (17.11%) and I (12.88%) scores.

**Figure 2:**
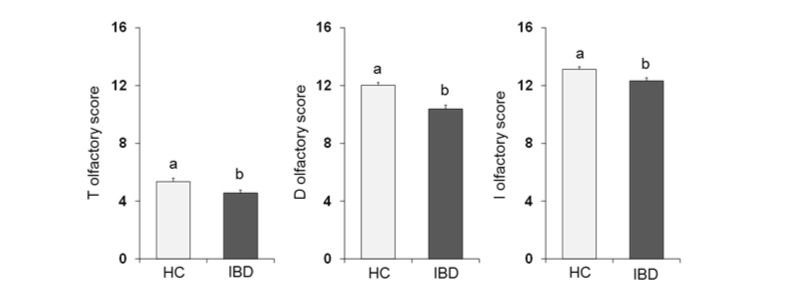
Mean (± SE) values of the T, D and I olfactory score determined in HC subjects (*n* = 99), and IBD patients (*n* = 100). Different letters (a and b) indicate a significant difference (T score: *p* = 0.015, Fisher’s LSD test subsequent one-way ANOVA; D score: *p* < 0.001; I score: *p* = 0.003; Duncan’s test subsequent one-way ANOVA).

One-way ANOVA (Figure 2) showed that the olfactory score obtained by HC subjects was significantly higher than that obtained by IBD patients for the T-test (*F*_1,197_ = 6.02; *p* = 0.015), D-test (*F*_1,197_ = 24.28; *p* < 0.001) and I-test (*F*_1,197_ = 8.88; *p* = 0.003).

Table 3 shows the distribution of the HC subjects and IBD patients classified as normosmic or hyposmic based on their Threshold (T), Discrimination (D) and Identification (I) olfactory status. Fisher’s method evidenced that the percentage of HC subjects who were classified as normosmic or hyposmic for their D olfactory status differed from that determined in IBD patients (*χ2* = 7.27, *p* = 0.007). Specifically, 85.86% (*n* = 85) and 14.14% (*n* = 14) of HC subjects were, respectively, normosmic or hyposmic, while in the case of IBD patients, 70% were classified as normosmic and 30% as hyposmic. No differences in percentage between subjects classified as normosmic or hyposmic on the basis of T and I olfactory status were found.

### 3.2. BMI Effects

**Figure 3:**
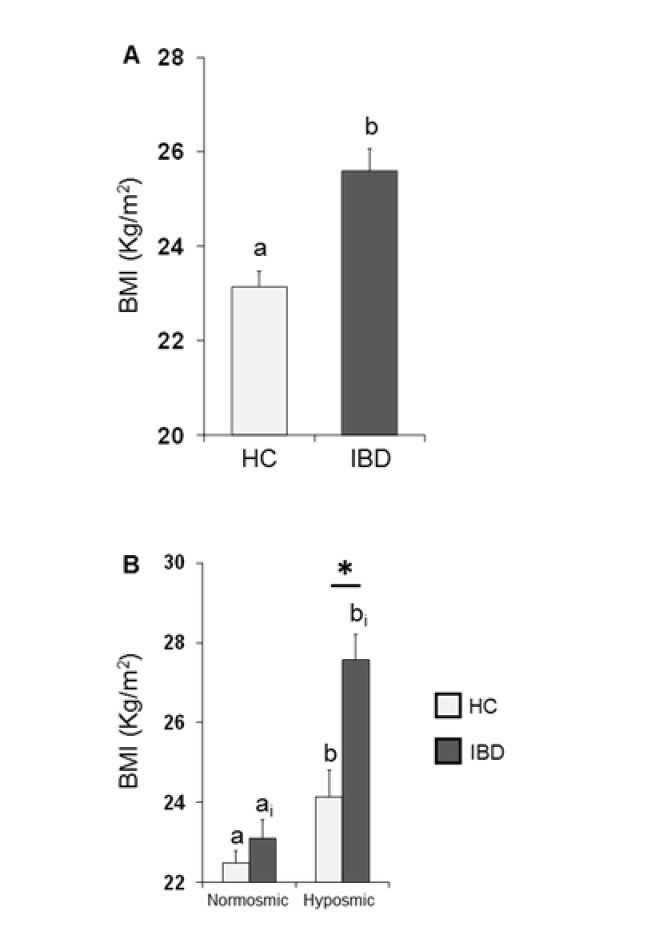
Mean (± SE) values of BMI determined in HC subjects (*n* = 99) and IBD patients (*n* = 100) (**A)** and according to their overall TDI olfactory status (B). (A) Different letters (a and b) indicate a significant difference between HC and IBD (*p* < 0.001; Fisher’s LSD test). (**B**) Different letters indicate a significant difference between normosmic and hyposmic individuals within the same population (HC subjects or IBD patients) (a and b for HC: TDI status *p* = 0.032; a_i_ and b_i_ for IBD: TDI status *p* < 0.001; Fisher’s LSD test). (*) indicates a significant difference between HC subjects and IBD patients within the same olfactory status (normosmic or hyposmic individuals) (*p* < 0.001; Fisher’s LSD test).

The mean values ± SE of BMI determined in HC subjects and IBD patients are shown in Figure 3A. One-way ANOVA revealed that the BMI of HC subjects was significantly lower than that of IBD patients (*F*_1,197_ = 18.44; *p* < 0.001). Figure 3B shows the same data according to their overall olfactory status (TDI status). Two-way ANOVA highlighted significant interactions of the health state × TDI olfactory status (*F*_1,195_ = 4.84, *p* = 0.029); post-hoc comparisons revealed that individuals who were hyposmic showed a BMI higher than those who were normosmic (HC *p* = 0.032; IBD *p* < 0.001; Fisher’s LSD test) and that IBD patients who were hyposmic had a higher BMI than hyposmic HC subjects (*p* < 0.001; Fisher’s LSD test), while no difference was observed between normosmic HC and IBD individuals (*p* > 0.05; Fisher’s LSD test).

### 3.3. Olfactory Function and Genotyping for OBPIIa Polymorphism, rs2590498 (A/G)

Molecular analysis revealed that HC subjects and IBD patients did not differ on the basis of their genotype distribution (*χ2*= 0.14, *p* = 0.935) and allele frequency (*χ2*= 0.16, *p* = 0.924) (Table 4).

**Figure 4:**
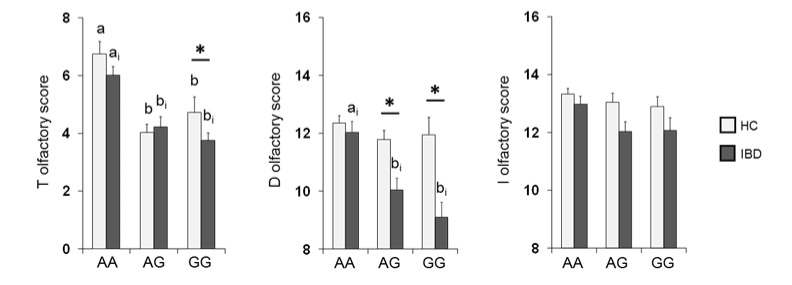
Mean (± SE) values of the T, D and I olfactory score according to genotypes of the *OBPIIa* locus determined in HC subjects (*n* = 99; 36 AA, 17 AG, 46 GG) and IBD (*n* = 100; 30 AA, 28 AG, 42 GG). Different letters indicate a significant difference: a-b for HC subjects (p < 0.001; Fisher’s LSD test); a_i_-b_i_ for IBD patients (T score *p* < 0.01, Fisher’s LSD test; D score *p* < 0.001, Duncan’s test). (*) indicates a significant difference with respect to the corresponding value of HC subjects (T score GG: 0.024, Fisher’s LSD test; D score AG: *p* < 0.001, GG: *p* < 0.001, Duncan’s test).

The mean values ± SE of the T, D and I scores obtained from HC subjects and IBD patients according to *OBPIIa* locus are shown in Figure 4. Two-way MANOVA revealed a significant interaction of the health state × *OBPIIa* genotypes on the T, D and I scores (*F*_6,382_ = 2.18; *p* = 0.04). In detail, pairwise comparisons showed that individuals who were homozygous for the major allele A exhibited T (both in HC subjects and IBD patients) and D scores (only in IBD patients) that were statistically higher than those of heterozygous individuals (T score: *p* < 0.01; D score: *p* < 0.001; Fisher’s LSD test) or homozygous for the minor allele G (T score: *p* < 0.001; D score: *p* < 0.001; Fisher’s LSD test). In addition, we found that IBD patients who were heterozygous or GG homozygous reached significantly lower T (*p* = 0.024; Fisher’s LSD test) and D (*p* < 0.001; Fisher’s LSD test) scores than HC subjects, and IBD patients who were heterozygous reached significantly lower D scores than HC subjects (*p* < 0.001; Fisher’s LSD test). No other differences between HC subjects and IBD patients according to the *OBPIIa* genotype were found. 

**Figure 5:**
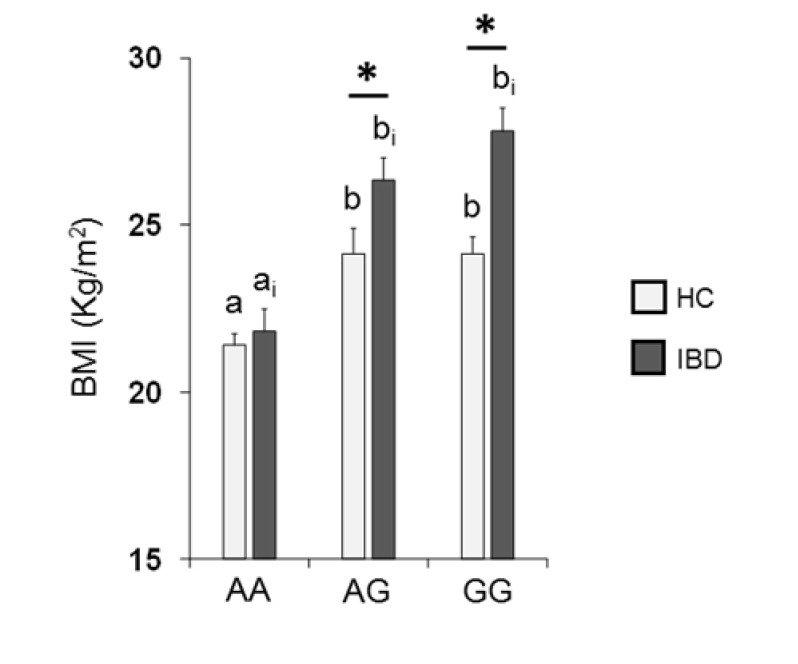
Mean (± SE) values of BMI according to genotypes of the *OBPIIa* locus determined in HC subjects (*n* = 99; 36 AA, 17 AG, 46 GG) and IBD patients (*n* = 100; 30 AA, 28 AG, 42 GG). Different letters indicate a significant difference: a-b for HC subjects (*p* < 0.01; Fisher’s LSD) test; a_i_-b_i_ for IBD patients (*p* < 0.001; Fisher’s LSD test). (*) indicates a significant difference with respect to the corresponding value of HC subjects (AG: *p* = 0.017; GG: *p* < 0.001).

Figure 5 shows the mean values ± SE of BMI determined in HC subjects and IBD patients according to the *rs2890498* polymorphism of the *OBPIIa* gene. Two-way ANOVA revealed a significant interaction of health state × *OBPIIa* genotype on BMI (*F*_2,193_ = 4.05, *p* = 0.018). For HC subjects, post-hoc comparisons indicated that subjects who were homozygous for the A-allele exhibited a lower BMI than subjects that were homozygous for the G-allele (*p* < 0.001; Fisher’s LSD test) or heterozygous (*p* = 0.008; Fisher’s LSD test). In addition, pairwise comparison revealed that IBD patients who were homozygous for the G-allele or heterozygous exhibited a higher BMI than patients that were AA homozygous (*p* < 0.001; Fisher’s LSD test). The BMI of HC subjects who had the GG or AG genotype was significantly lower than that of IBD patients with the same genotype (GG genotype: *p* < 0.001; AG genotype: *p* = 0.017; Fisher’s test LSD).

## 4. Discussion

The main goal of this study was to evaluate the olfactory function of IBD patients and compare it with that of a group of healthy control (HC) subjects. The results we obtained show that the general olfactory sensitivity of IBDs is significantly impaired when compared with HC subjects, as shown by the lower performance they reached in the total TDI score and by the increased number of hyposmic subjects. Since the TDI olfactory status depends on the abilities of olfactory threshold, odor discrimination and odor identification, we investigated which of them were compromised. The results we obtained show that IBD patients reached T, D and I values that were significantly lower than those obtained by HC subjects. We also found a significantly higher number of hyposmic subjects for the D olfactory status (30% of IBD patients were hyposmic compared to 14% of HC subjects), in accordance with the fact that the D score is the main contributor to the total TDI score obtained by IBD patients. The lower olfactory threshold scores (which means that individuals showed a higher olfactory threshold) we observed in IBD patients, as compared to controls, can be explained by the elevated levels of tumor necrosis factor (TNF-α) which were identified in the blood and intestinal mucosa of IBD patients. High levels of TNF-α have been reported to cause the loss of mature olfactory sensory neurons (OSNs) in the olfactory epithelium [45,68,69], thus affecting the olfactory threshold which, to some degree, represents the peripheral olfactory function [70,71,72]. The relationship between elevated TNF-α levels and olfactory function is supported by the data in Supplementary Figure S1, which show that HC subjects reached T olfactory scores significantly higher than IBD patients not treated with anti-TNF-α, while no difference was observed in IBD patients treated with the same drug. 

While a threshold impairment has already been observed in a previous study on the olfactory performance of IBD patients [36], these are the first results highlighting an impairment in odor discrimination and odor identification, higher-order olfactory functions, that require a more pronounced involvement of cognitive factors [70,71]. IBD patients sometimes show depression [73], and some of the altered brain areas in depressed patients are essential for the processing of olfactory information [74,75,76]. Additionally, previous studies have shown a relationship between deficits in D and I olfactory ability and depression [13,77,78]. However, since none of the patients recruited for this study were on antidepressant therapy, future studies will be needed in order to better understand the relationship between IBD and impairment in odor discrimination and odor identification.

The second aim of our study was to compare the body weight of IBD patients (by calculating the BMI) with that of HC subjects, also in relation to their olfactory status. In fact, olfactory sensitivity plays a role in food choices and intake, and its impairment affects eating behavior [2,12,19,70]. Individuals with olfactory dysfunction tend to experience a lower reward from food, report that food is less flavorful and less enjoyable, and compensate for this deficiency by changing their feeding habits (e.g., eating saltier, sweeter, more spicy foods) and by decreasing their intake of low-fat foods [12,15,30]. A similar eating behavior has been observed in IBD patients, who increase their consumption of sucrose and refined carbohydrates and reduce that of fruits and vegetables [79,80]. Both individuals with olfactory dysfunction and IBD patients may display variations in body weight [12,30,36,59]. The results we obtained show that: first, the BMI of hyposmic IBD patients was significantly higher than that of hyposmic HC controls, while no statistical difference was found between normosmic IBD and normosmic controls; second, the BMI of normosmic was lower than that of hyposmic individuals in both HC subjects and IBD patients. Based on these findings, we speculate that olfactory impairment may be considered a more important factor than the disease in causing an increase in BMI. These results are in agreement with previous studies that have shown that obese adults show reduced olfactory sensitivity [81] and that a negative correlation exists between body weight and orthonasal olfactory ability [82]. The sense of smell participates in the cephalic phase responses to food, which play a direct role in regulating meal size [83], by acting on appetite and satiety [1,15,19,84]. Several studies have shown that a reduced olfactory sensitivity determines a reduced response of the cephalic phase, with a consequent delay in reaching the sense of satiety and an increase in the duration of the meal: this leads to an over-feeding of gratifying and palatable foods that causes an increase in body weight [85,86,87]. Finally, the expression of olfactory receptors (ORs) has been found in the enterochromaffin cells of the gut, and their activation by odors leads to the release of serotonin that affects gut motility and increases satiety [88,89]; this means that an increase in their threshold leads to a lower release of serotonin, a reduced sense of satiety, a higher intake of calories and longer duration of meals [36]. 

Individuals exhibit a physiological variability in their olfactory function due to environmental and genetic factors [20,21,22,23,24]. Recent studies on different groups of healthy subjects have shown that this variability can be, at least partially, determined by the *rs2590498* (A/G) polymorphism of the *OBPIIa* gene, both in terms of the ability to perceive complex odors and single molecules [24,60,61]. In particular, these authors found that individuals who were homozygous for the major allele A showed a lower olfactory threshold than heterozygotes and homozygotes for the minor allele G. The results of this study show that both IBD patients and HC subjects who were homozygous AA achieved higher T-scores than heterozygous and homozygous GG and surprisingly, among IBD patients, those with the AA genotype also achieved higher scores than those with genotype AG and GG in the D-test. These results are in agreement with previous studies that report that OBPs play an important role both in carrying odorous molecules, generally lipophilic, through the mucus layer to the ORs, and in odor discrimination [90,91,92,93]. Patients with GG genotype reached a significantly lower T-score than controls with the same genotype, while no difference was observed between patients and controls with at least one A allele. This suggests that the lowest T-score obtained by patients, as shown in Figure 2, is mainly determined by GG homozygous ones. Additionally, the D olfactory performances of IBD patients carrying two sensitive alleles (AA) were not different from those of HC subjects. Similarly, recent evidence has shown that women with Parkinson’s Disease (PD) who are AA homozygous exhibit a better olfactory performance than heterozygous or GG homozygous PD women and that their olfactory scores are not different from those of HC subjects [94]. Finally, we found that both controls and patients who were homozygous for the A-allele exhibited a lower BMI than individuals who were homozygous for the G-allele or heterozygous, and patients with at least one G-allele showed a higher BMI than controls. Based on these findings, we can hypothesize the following: (a) in IBD patients, the presence of at least one sensitive allele (A) prevents the decline in the olfactory threshold compared to controls; (b) the presence of at least one G allele, on the one hand, is sufficient to impair odor discrimination, and on the other, it increases the BMI of patients compared to controls.

## 5. Conclusions

In conclusion, our findings show an inverse relationship between olfactory function and BMI in IBD patients. This may be partially explained by a delay in the satiety sensation and by an increase in meal duration, resulting in over-feeding on gratifying and palatable foods. In addition, the AA genotype for *OBPIIa*, as shown in this study for the first time, appears to protect IBD patients from both olfactory dysfunctions and BMI increases. AA individuals may better tolerate a nutritional therapy aimed at reducing the consumption of foods rich in fats and proteins, as possible treatment to improve their quality of life. Therefore, further studies will be needed to better understand the relationship between olfactory function, food selection and tolerance to nutritional therapy in IBD patients.

## Figures and Tables

**Table 1:** Distribution of the healthy control (HC) subjects and inflammatory bowel disease (IBD) patients classified as normosmic or hyposmic based on their overall olfactory status (TDI).

	Group	HC	IBD	*p*-Value
Variable	Olfactory status	*n* (%)	*n* (%)	
TDI	Normosmic	60 (61)	44 (44)	0.019
	Hyposmic	39 (39)	56 (56)	

*p*-value derived from Fisher’s Exact Test. HC subjects (*n* = 99), IBD patients (*n* = 100).

**Table 2:** Stepwise forward multiple regression models for TDI score in HC subjects (*n* = 99) and IBD patients (*n* = 100).

Populations	Variable	Overall Model		Parameter Estimate		Each Step
		(adj R^2^)	(*p*)	(sr)	(*p*)	(R^2^)
HC	T D I	0.9949	<0.00001	0.5709 0.4576 0.3930	<0.00001 <0.00001 <0.00001	0.5496 0.8372 0.9949
IBD	D T I	0.9999	<0.00001	0.4088 0.3798 0.3553	<0.00001 <0.00001 <0.00001	0.7033 0.8711 0.9999

Independent variables included: Threshold (T), Discrimination (D) and Identification (I) score. Adj = adjusted; sr = semipartial correlation.

**Table 3:** Distribution of the healthy control (HC) subjects and inflammatory bowel disease (IBD) patients classified as normosmic or hyposmic based on their Threshold (T), Discrimination (D) and Identification (I) olfactory status.

	Group	HC	CD	*p*-Value
Variable	Olfactory Status	*n* (%)	*n* (%)	
T	Normosmic	57 (57.58)	65 (65.00)	0.282
	Hyposmic	42 (42.42)	35 (35.00)	
D	Normosmic	85 (85.86)	70 (70.00)	0.007
	Hyposmic	14 (14.14)	30 (30.00)	
I	Normosmic	91 (91.92)	88 (88.00)	0.358
	Hyposmic	8 (8.08)	12 (12.00)	

*p*-value derived from Fisher’s Exact Test. HC subjects (*n* = 99), IBD patients (*n* = 100).

**Table 4:** Genotype distribution and allele frequency of the *rs2590498* polymorphism of the *OBPIIa* gene (A/G) in HC subjects and IBD patients.

TDI	HC *n* (%)	IBD *n* (%)	*p*-Value
*Genotype*			
AA AG GG	36 (36.36) 17 (17.17) 46 (46.47)	30 (30.00) 28 (28.00) 42 (42.00)	0.935
*Allele*			
A G	89 (44.95) 109 (50.05)	88 (44.00) 112 (56.00)	0.924

*p*-value derived from Fisher’s Exact Test. HC subjects (*n* = 99), IBD patients (*n* = 100).

## Data Availability

The data presented in this study are available on request from the corresponding author. The data are not publicly available due to restrictions, e.g., privacy or ethical.

## Supplementary Materials

The following are available online at https://www.mdpi.com/2072-6643/13/2/703/s1, Figure S1: Mean (± SE) values of the T olfactory score determined in HC subjects (*n* = 99), and IBD patients not treated with anti-TNF-α (**A**; *n* = 78) or IBD patients treated with anti-TNF-α (**B**; *n* = 22). One-way ANOVA revealed a significant effect of the health status on the T olfactory score in the IBD patients treated with anti-TNF-α (*F*_1,175_ = 6.02; *p* = 0.005), while no difference was observed between HC subjects and IBD patients not treated with anti-TNF-α(*F*_1,119_ = 0.02; *p* = 0.889) α. Different letters indicate a significant difference (*p* = 0.005, Fisher’s LSD test).
