# Supplementary material for: Olfactory Function in Patients with Inflammatory Bowel Disease (IBD) Is Associated with Their Body Mass Index and Polymorphism in the Odor Binding-Protein (OBPIIa) Gene
Source: Nutrients. 2021 Feb 22;13(2):703. doi: 10.3390/nu13020703 (PMC7926749; doi:10.3390/nu13020703)
Supplement: Supplementary file 1 [file nutrients-13-00703-s001.zip › nutrients-1105233-supplementary.pdf]

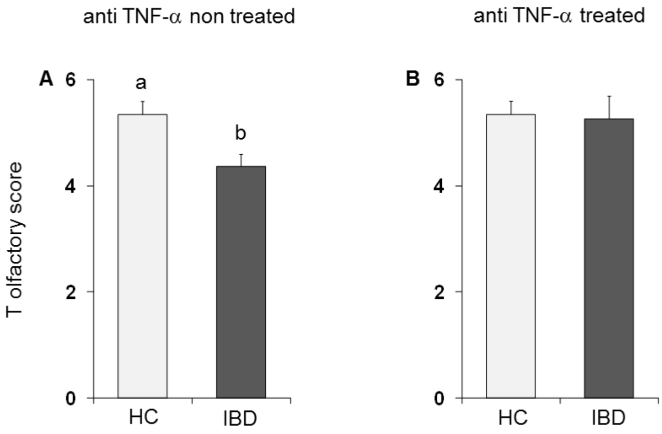

Figure S1. Mean (± SE) values of the T olfactory score determined in HC subjects (n = 99), and IBD patients not treated with anti TNF-α (**A**; n = 78) or IBD patients treated with anti TNF-α (**B**; n = 22). One-way ANOVA revealed a significant effect of the health status on the T olfactory score in the IBD patients treated with anti TNF-α ($F_{1,175}$ = 6.02; p = 0.005), while no difference was observed between HC subjects and IBD patients not treated with anti TNF-α ($F_{1,119}$ = 0.02; p = 0.889). Different letters indicate a significant difference (p = 0.005, Fisher's LSD test).
